# Conversion therapy from unresectable stage IIIC non-small-cell lung cancer to radical surgery *via* anti-PD-1 immunotherapy combined with chemotherapy and anti-angiogenesis: A case report and literature review

**DOI:** 10.3389/fonc.2022.954685

**Published:** 2022-09-14

**Authors:** Guohua Jia, Shuimei Zhou, Tangpeng Xu, Yabing Huang, Xiangpan Li

**Affiliations:** ^1^ Cancer Center, Renmin Hospital of Wuhan University, Wuhan, China; ^2^ Department of Blood Transfusion, Zhongnan Hospital of Wuhan University, Wuhan, China; ^3^ Department of Pathology, Renmin Hospital of Wuhan University, Wuhan, China; ^4^ Department of Oncology, Renmin Hospital of Wuhan University, Wuhan, China

**Keywords:** locally advanced, unresectable lung cancer, conversion therapy, squamous cell carcinoma, immunotherapy

## Abstract

The prognosis of patients with stage IIIC non-small-cell lung cancer (NSCLC) is poor due to the loss of surgical treatment opportunities. Improving the prognosis of these patients with IIIC NSCLC urgently needs to be addressed. Here, we report a stage IIIC (T4N3M0 IIIC (AJCC 8th)) NSCLC patient treated with 2 cycles of anti-PD-1 immunotherapy combined with chemotherapy and anti-angiogenesis therapy; after two cycles of treatment, the patient achieved a partial response and obtained the opportunity for surgical treatment. After the operation, the patient achieved a pathological complete response and successfully transformed from unresectable stage IIIC lung cancer to radical surgery (ypT0N0M0). Our study is expected to provide new ideas for treating patients with unresectable stage IIIC NSCLC in the future.

## Introduction

Lung cancer has a high incidence rate and mortality; surgical treatment is an essential radical cure for lung cancer. However, only I-IIIA stage and partial IIIB stage non-small-cell lung cancer (NSCLC) patients can obtain the chance of complete surgical resection, and the five-year survival rate is 36%-92%. Approximately 50% of IIIC stage and distant metastasis NSCLC patients have a poor quality of life and short survival time (1–3). In recent years, due to the application of new antitumor drugs, such as immunotherapy and targeted agents, the overall survival (OS) and quality of life have been effectively improved in patients with locally advanced and distant metastatic NSCLCs. Neoadjuvant chemotherapy also significantly improved the prognosis of patients with stage IB-IIIA lung cancer (4–6). However, for locally advanced lung cancer patients with unresectable stage IIIC lung cancer, the 5-year OS remains suboptimal (7, 8). Improving the survival time and quality of life of those patients needs to be further explored. This study reported a case of unresectable stage IIIC NSCLC that achieved pathological complete response (pCR) and obtained the opportunity for complete resection after 2 cycles of anti-PD-1 immunotherapy combined with chemotherapy and anti-angiogenesis therapy. We also reviewed relevant works in the literature.

## Case presentation

**Figure 1:**
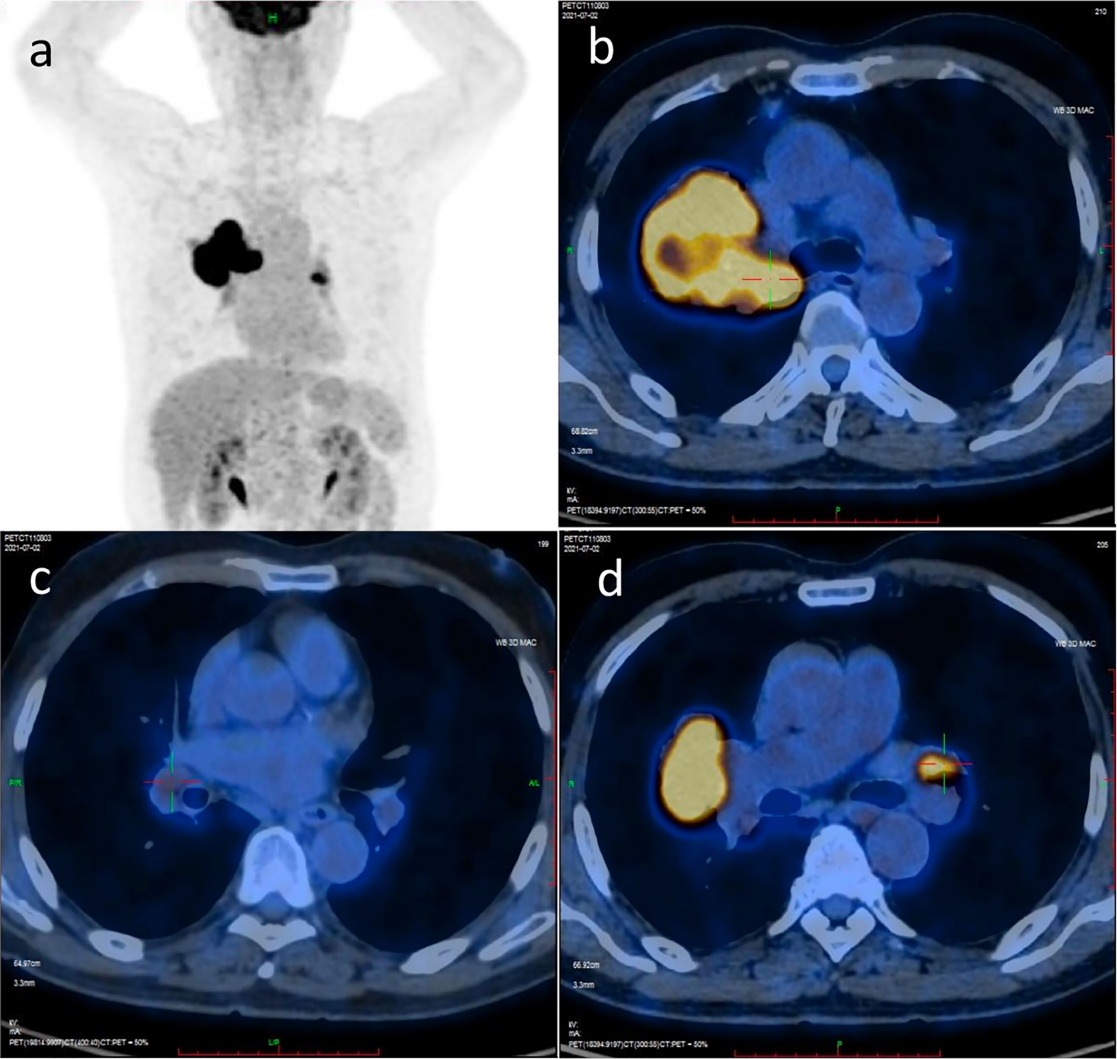
PET/CT scan image before treatment. **(A)** PET/CT scan image of chest and abdomen; **(B)** PET/CT scan image of irregular soft tissue mass in the right hilum; **(C)** PET/CT scan image of the enlarged lymph node in right hilar; **(D)** PET/CT scan image of the enlarged lymph node in left hilar.

**Figure 2:**
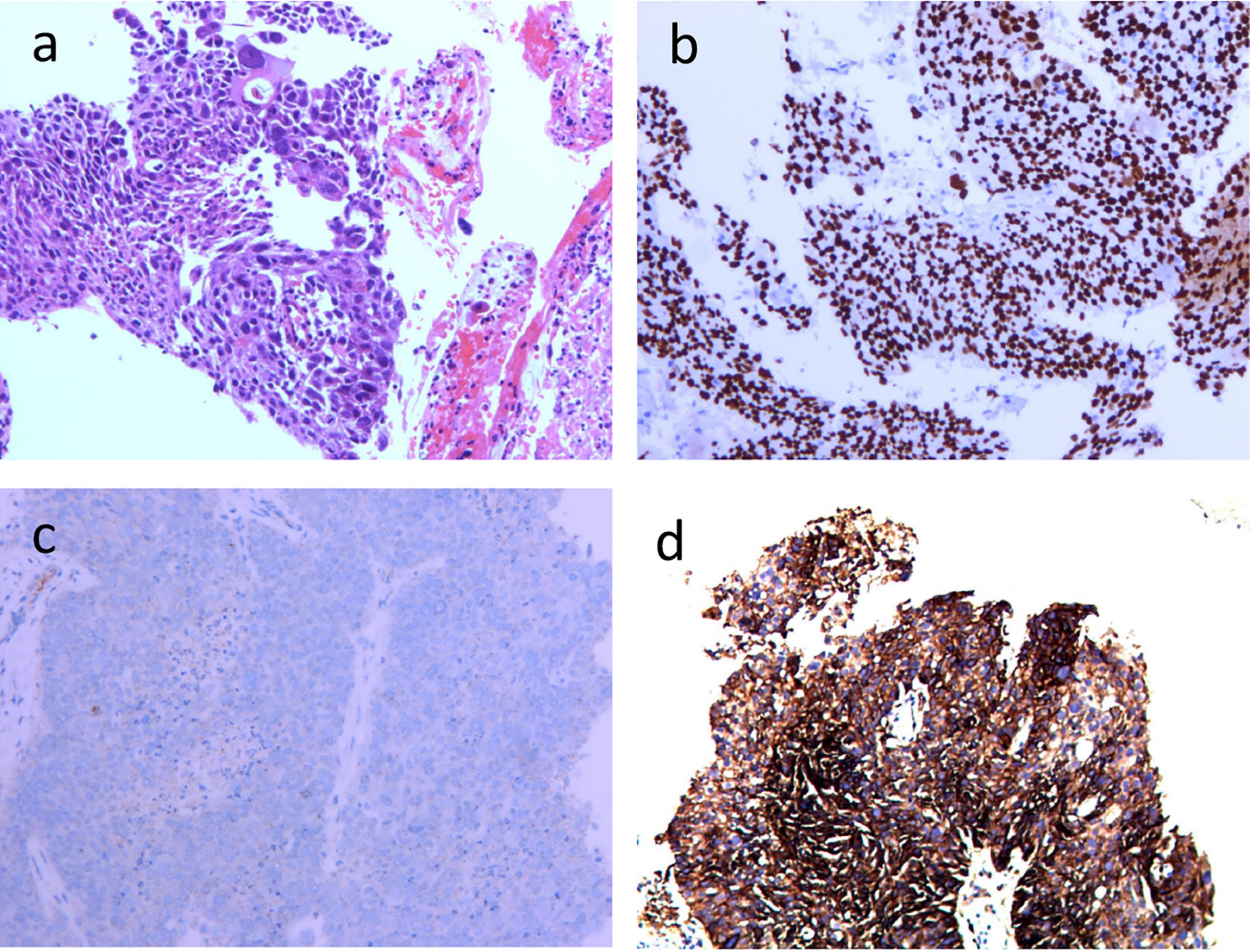
Histopathological and immunohistochemical examinations of primary tumor focus in the right lung before operation (100×) **(A)** HE-stained image of primary tumor focus; **(B)** p40 (+); **(C)** ALK (-); **(D)** PD-L1 (+).

A 74-year-old male patient visited our hospital on June 29, 2021, with the chief complaint of “finding a pulmonary mass for over 3 months”. The PET-CT showed a size of 7.7 cm × 6.8 cm × 5.7 cm irregular soft tissue mass in the right hilum, standardized uptake value (SUV) max: 14.0; enlarged lymph node in the right hilar with a size of 1.6 cm × 1.2 cm, SUVmax 3.6; enlarged lymph node in the left hilar with a size of 1.9 cm × 1.3 cm, SUVmax 9.2; which indicated that the patient suffered from right lung cancer with bilateral hilar lymph node metastasis (**Figure 1**). Brain enhanced MRI showed no signs of intracranial metastasis. Then, percutaneous transthoracic needle biopsy of the pulmonary mass under CT guidance was performed. The pathological results showed NSCLC, most likely lung squamous cell carcinoma. Immunohistochemistry: CK5/6 (+), p63 (+), P40 (+), p53 (-, mutant), Ki67 (+), approximately 50%, CK7 (-), TTF-1 (-), CD56 (-), ALK (-), programmed cell death ligand 1 (PD-L1) expression was positive, tumor Proportion Score: 83% (**Figure 2**). Molecular pathologic analysis results showed that EGFR mutations and ALK rearrangement were negative. The patient was diagnosed with stage IIIC [cT4N3M0 (AJCC 8th)] lung squamous cell carcinoma based on imaging examination and pathological results.

**Figure 3:**
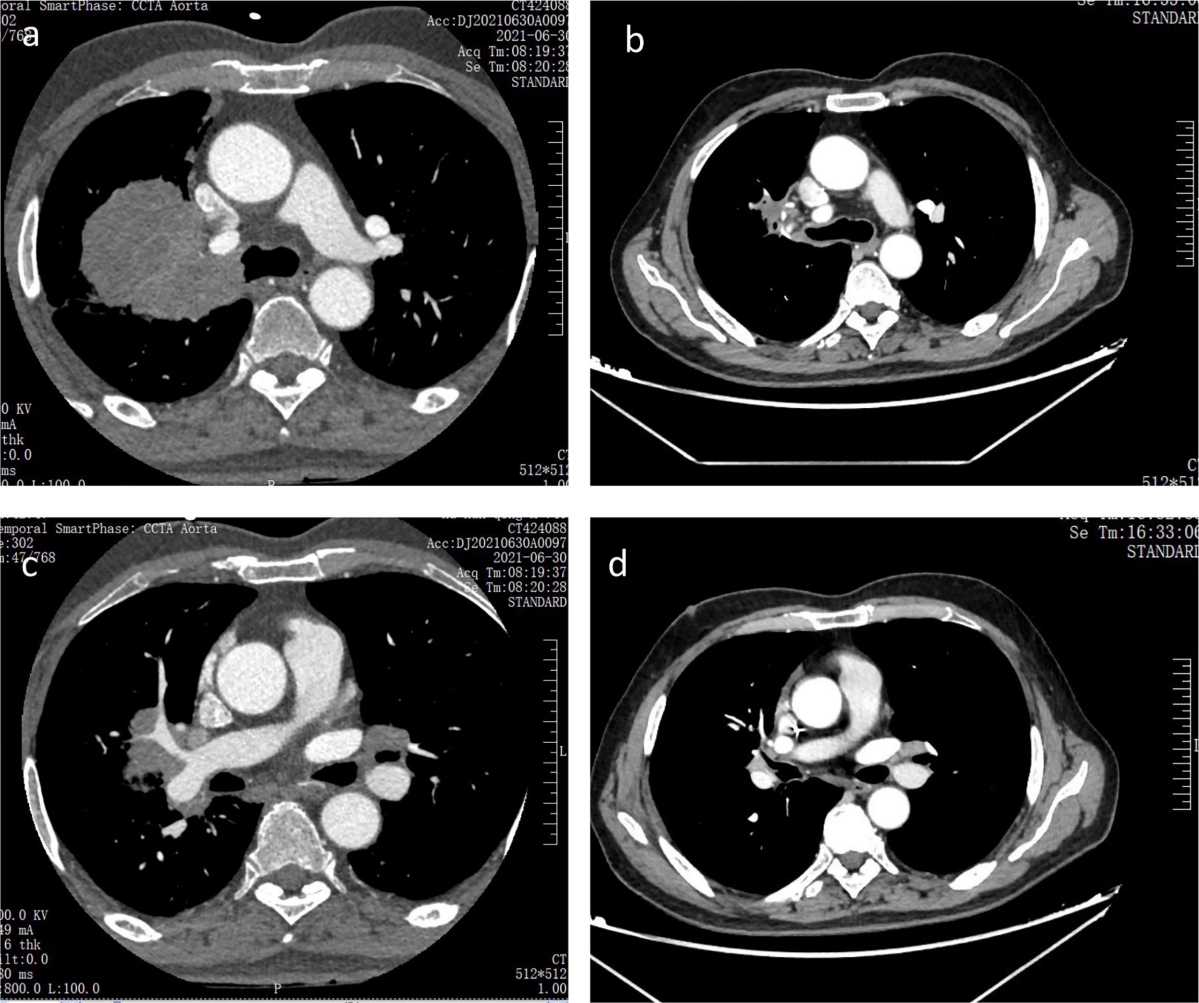
Chest CT scans before and after two cycles of anti-PD-1 immunotherapy combined with anti-angiogenesis therapy and chemotherapy **(A, B)** Chest CT scans of primary tumor focus in the right lung before and after two cycles of anti-PD-1 immunotherapy combined with anti-angiogenesis therapy and chemotherapy respectively; **(C, D)** Chest CT scans of the metastatic lymph node in left hilar before and after two cycles of anti-PD-1 immunotherapy combined with anti-angiogenesis therapy and chemotherapy respectively.

**Figure 4:**
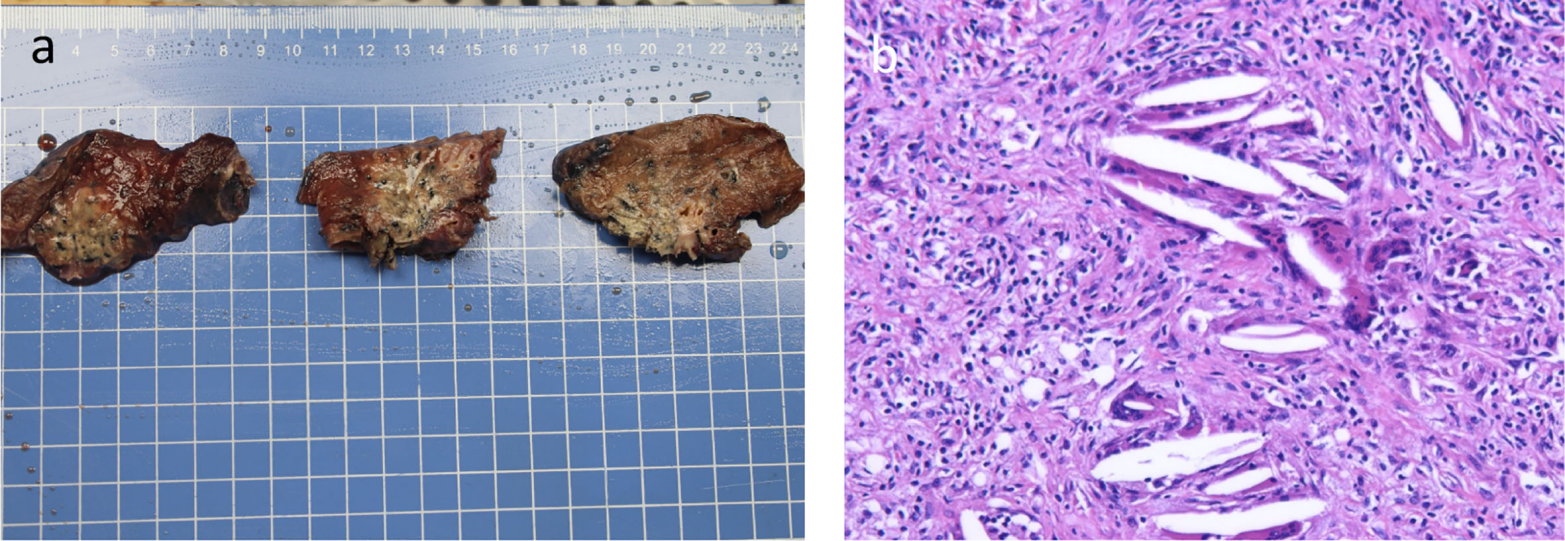
Surgical tissue specimen and postoperative histopathological HE-stained picture of the tumor tissue **(A)** Surgical tissue specimen; **(B)** HE-stained image of resected tumor tissue (100×).

**Figure 5:**
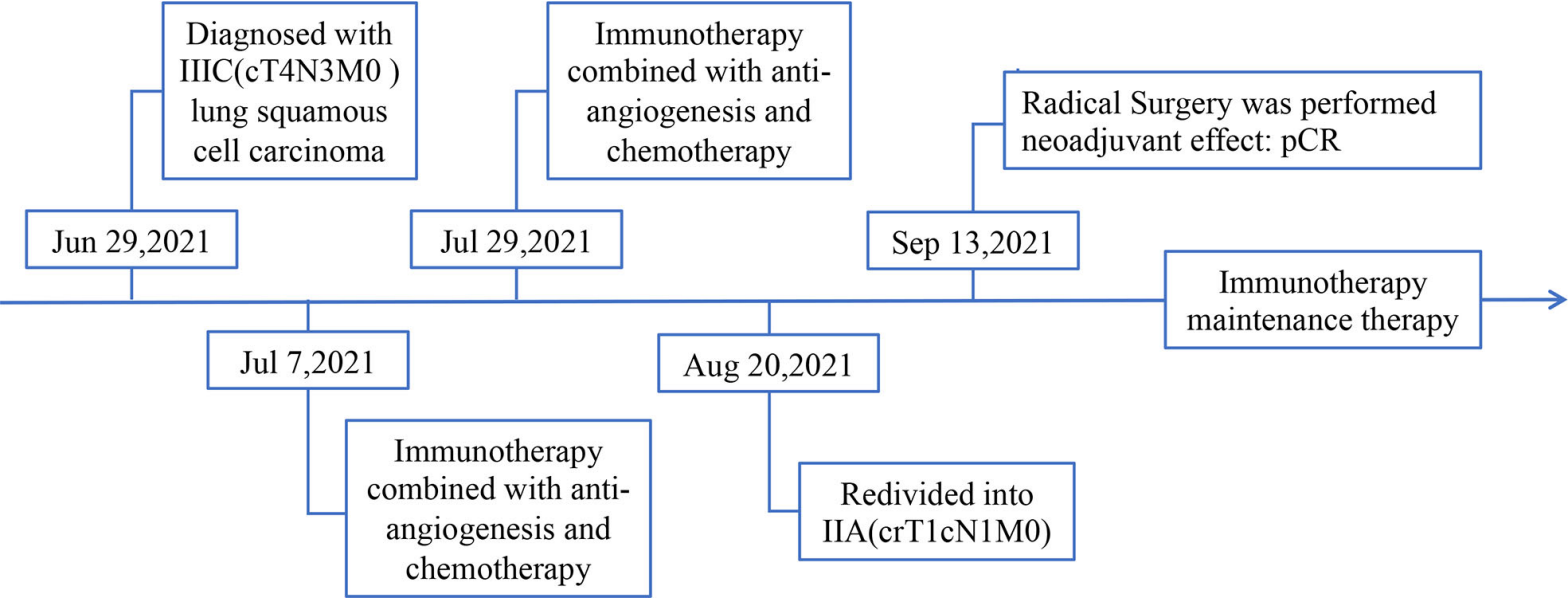
Diagnosis and treatment information of this patient.

Then, on July 7, 2021, and July 29, 2021, the patient received 2 cycles of anti-PD-1 immunotherapy (tirelizumab 200 mg, q3w) combined with chemotherapy (lobaplatin 60 mg, q3w and albumin paclitaxel 400 mg, q3w) and antiangiogenic therapy (recombinant human endostatin 180 mg, q3w), respectively. After 2 cycles of therapy, the chest and abdomen enhancement CT showed the following: 1. The neoplastic lesions in the upper lobe of the right lung were smaller than before, with a size of approximately 2.3 cm × 2.6 cm. The enlarged lymph nodes in the mediastinum and hilar were smaller than before. The efficacy was evaluated as partial response (PR), and the clinical stage was redivided into IIA (crT1cN1M0) according to the chest and abdomen enhancement CT image, which opened up the opportunity for surgical treatment (**Figure 3**). The patient reported no level 3 or worse adverse events during previous treatment. On September 13, 2021, “lobectomy (right middle-upper lobectomy) + Brachioplasty + thoracoplasty + pleural adhesion lysis” was performed for this patient. The postoperative pathological examination showed that the primary cancer lesion was located in the right middle and upper lung lobe without tumor thrombus and nerve invasion. Anastomotic margin and the broken bronchial end were negative. The neoadjuvant effect of the primary tumor was as follows: total tumor collection: 0% of live tumor cells, 5% of necrosis, and 95% of stroma (fibrosis and inflammation). Residual tumor load grade: pCR. Lymph nodes sent for examination (Groups 2, 4, 7, 9, 10, 11, and 12) (0/5, 0/4, 0/5, 0/1, 0/6, 0/3, 0/2) showed no cancer metastasis. Additionally, no cancer metastasis was found in the parabronchial lymph nodes (0/1), which indicated that this patient had successfully transformed from unresectable stage IIIC lung cancer to radical surgery (ypT0N0M0) (**Figure 4**). To date, the patient was in follow-up with maintenance immunotherapy after the operation, and related examination showed no sign of relapse or metastasis. The related diagnosis and treatment information of this patient are shown in **Figure 5**.

## Discussion

This was a T4N3M0 stage IIIC (AJCC 8th) NSCLC patient who was unresectable according to the latest NCCN guide; however, after 2 cycles of anti-PD-1 immunotherapy combined with chemotherapy and anti-angiogenesis therapy, the primary tumor and metastatic lymph nodes were significantly reduced, and a radical surgical treatment opportunity was obtained. The postoperative pathological results showed that neoadjuvant treatment efficacy evaluation was pCR; we successfully transformed this patient into an early-stage lung cancer lesion (ypT0N0M0) *via* anti-PD-1 immunotherapy combined with chemotherapy and anti-angiogenesis, which may be of great significance in improving the prognosis of unresectable stage IIIC lung cancer patients.

Approximately 20% to 30% of NSCLC patients are classified as stage III, and treatment for stage III NSCLC is complex and controversial (9, 10). Neoadjuvant chemotherapy before surgery and adjuvant chemotherapy or definitive concurrent chemoradiotherapy after the operation were the standard therapies for resectable IIIA and partial stage IIIB patients (8, 11). For unresectable stage IIIB and IIIC NSCLC patients, multiple therapeutic measures were taken to prolong the lifespan of these individuals. The PACFIC trial showed that for unresectable stage IIIB and IIIC NSCLC patients, those who received durvalumab after chemoradiotherapy had a median OS of 47.5 months, and almost half of those patients lived longer than 4 years. Based on this evidence, platinum-based chemoradiotherapy following durvalumab treatment was recommended as first-line treatment for unresectable stage IIIB and IIIC NSCLC patients without EGFR or ALK mutations (12). Despite unresectable stage IIIB and IIIC NSCLC patients receiving survival benefits from the PACIFIC trial, unresectable locally advanced lung cancer patients still have a poorer prognosis than resectable lung cancer patients. Further improving the prognosis of unresectable locally advanced lung cancer patients is a great challenge, and new antitumor drugs, such as immunotherapy, might be viable options.

Although checkpoint blockade immunotherapy has played a significant role in improving the OS of lung cancer patients, the objective response rate of single immunotherapy was only approximately 20% in lung cancer patients; therefore, single immunotherapy was not recommended in lung cancer patients with PD-L1<50% (13, 14). Multiple studies have shown that there is crosstalk between chemotherapy and immunotherapy. MHC class I chain-related molecule A and B expression was associated with better prognosis for lung cancer patients. The cytotoxic drug cisplatin could upregulate MHC class I chain-related molecule A and B expression in NSCLC cell lines, antigen-presenting cells and dendritic cells, increase the infiltration of CD4+ and CD8+ T cells and NK cells, and boost the secretion of IFN-γ and TNF-α, thus modulating the tumor immune microenvironment (15–17). Additionally, patients treated with cytotoxic drugs had a higher ratio of effector T cells (Teffs) to Tregs and increased IL-2 levels (18, 19). In addition, cytotoxicity enhanced the expression of the T-cell costimulatory ligands CD70, CD80, and CD86 while stimulating tumor infiltration of APCs, thus improving the antitumor effects (20). PD-L1 expression was an efficacy predictive index for immunotherapy in cancer patients. Ludovic (21) and Li Zhou (22) found that cisplatin could upregulate PD-L1 staining in both tumor and immune cells in NSCLC mouse models and cohorts of patients. This evidence indicated that chemotherapy could modulate the tumor immune microenvironment to transform the “cold” tumor into a “hot” tumor, thus enhancing the treatment effect of immunotherapy.

The emergence of immune checkpoint inhibitor anti-PD-1 therapy has brought significant changes to the treatment of cancer patients. Many clinical studies, such as KEYNOTE-024, KEYNOTE-042, IMPOWER150, and KEYNOTE-189, have shown that anti-PD-1 immunotherapy alone or combined with other antitumor therapies, such as targeted therapy or chemotherapy, exhibits significant effects in the treatment of locally advanced and advanced NSCLC (23–26). Keynote 024 and Keynote 042 showed that compared with platinum-based chemotherapy, pembrolizumab monotherapy could significantly improve the OS of untreated advanced or metastatic NSCLC patients with PD-L1 positivity without EGFR or ALK alterations (23, 24, 27). Keynote 189 aimed to explore the efficacy and safety of first-line pembrolizumab plus pemetrexed-platinum in patients with metastatic nonsquamous NSCLCs. The results showed that in metastatic nonsquamous NSCLC patients, combined therapy significantly improved the prognosis, including OS and progression free survival, despite the expression of PD-L1 (25). The IMPOWER150 study included 1202 patients diagnosed with stage IV metastatic nonsquamous NSCLC who had not received chemotherapy before; the results showed that the median OS of atezolizumab and chemotherapy with or without bevacizumab was improved compared with chemotherapy alone (28). This evidence showed that immunotherapy had become an indispensable standard therapy for advanced or metastatic NSCLC patients with wild-type EGFR and ALK genes.

In addition to unresectable advanced or metastatic NSCLC patients, anti-PD-1 immunotherapy has also demonstrated critical progress in the neoadjuvant treatment of patients with resectable IB-IIIB NSCLC. At present, the main modes of neoadjuvant immunotherapy are neoadjuvant monoimmunotherapy, neoadjuvant double immunotherapy, and neoadjuvant immune combined with chemotherapy (29, 30). LCMC3 is a phase II study that explored the efficacy of neoadjuvant monoimmunotherapy in resectable stage IB-IIIB NSCLC patients. The results showed that 43% of the patients achieved downstaging, 7% achieved pCR, and 92% achieved R0 resection (31). After neoadjuvant monoimmunotherapy achieved positive results, NEOSTAR research results showed that compared with neoadjuvant monoimmunotherapy, neoadjuvant double immunotherapy had a higher major pathological response (MPR) (50% vs. 24%) and pCR rate (38% vs. 10%), suggesting that neoadjuvant monoimmunotherapy might be insufficient (32). Compared with single neoadjuvant immunotherapy or double immunotherapy, more attention is given to the treatment of neoadjuvant immunotherapy combined with chemotherapy. Checkmate-159 is an open-label, multicenter, single-arm clinical study exploring the safety and efficacy of neoadjuvant immunotherapy combined with chemotherapy in resectable NSCLC. Twenty-three patients over 18 years old with clinically diagnosed stage IIA-IIIB NSCLC were enrolled in the study. After receiving 3-4 cycles of neoadjuvant immunotherapy combined with chemotherapy, the subjects underwent surgery. The study showed that the overall effective rate was 73.9%, including 73.9% of patients with PR and 26.1% of patients with stable-disease, of which 50% reached MPR and 30% reached pCR (33). NADIM is an open-label, multicenter, single-arm phase II clinical study. Forty-six patients with stage IIIA NSCLC were included in the study. The subjects underwent 3 cycles of neoadjuvant immunotherapy and chemotherapy, and postoperative immunotherapy was maintained for one year. The results indicated that 76% of patients showed ORR with 72% PR and 4% complete-response, and no patients had disease progression during neoadjuvant therapy. In terms of progression free survival, patients with neoadjuvant efficacy evaluation of MPR and pCR were significantly better with efficacy evaluation of PR and stable-disease (34). Catherine, a Shu (35) reported that after treatment with atezolizumab plus carboplatin and paclitaxel, 57% of patients with resectable stage IB-IIIA NSCLC showed MPR, and 33% of patients achieved pCR; in particular, 50% of patients with N2-lymph nodes achieved a descending stage. Although the above studies are single-arm studies, the addition of neoadjuvant immunotherapy significantly improves the efficacy of neoadjuvant therapy compared with previous neoadjuvant chemotherapy, with only a 5-8% pCR rate (36–38). CheckMate 816 is a phase III trial, and 358 resectable IB-IIIA NSCLC patients were randomly divided into a chemotherapy arm or a nivolumab plus chemotherapy group. Surgery was performed after 3 cycles of treatments. Results showed that more patients in the nivolumab plus chemotherapy group received radical surgery than in the chemotherapy group (83.2% vs. 77.8%), and the pCR rate in the nivolumab plus chemotherapy group was 10 times higher than that in the chemotherapy group (24.0% vs. 2.2%). Nivolumab plus chemotherapy group patients also had longer event-free survival than chemotherapy alone (31.6 months vs. 20.8 months) without causing more adverse events (39). Results of representative neoadjuvant immunotherapy clinical trials in resectable NSCLC were shown in **Table 1**. More phase III clinical studies of neoadjuvant immunotherapy, such as IMPOWER 030, are ongoing; we hope that these studies will provide more precise evidence for neoadjuvant immunotherapy in lung cancer. In this case, we applied two cycles of neoadjuvant immunotherapy combined with chemotherapy and anti-angiogenesis to successfully transform this unresectable stage IIIC lung cancer patient into radical surgery (ypT0N0M0), which significantly improved this unresectable stage IIIC lung cancer patient’s prognosis. In terms of the safety of neoadjuvant immunotherapy, most clinical research results show that the side effects of neoadjuvant immunotherapy are slight and well tolerated. Similarly, this patient showed no grade 3-4 adverse reactions during treatment, but there were no related data about adverse events of immunotherapy in preoperative IIIC patients. We still need to be vigilant for possible or unpredictable adverse events before the safety of conversion immunotherapy can be confirmed by large-scale phase III clinical studies. Presently, adjuvant therapy after neoadjuvant immunotherapy is still inconclusive. According to the patient’s adverse reactions, clinical evaluation, adjuvant immunotherapy, adjuvant chemotherapy with or without radiotherapy were optional adjuvant therapy. As for this case, he had a good treatment effect to immunotherapy and without adverse reactions of immunotherapy, moreover, he had a PD-L1 tumor Proportion Score ≥ 50%, which indicated he might benefit more from adjuvant immunotherapy. Therefore, according to clinical evaluation and relevant literatures, 12 months adjuvant immunotherapy were prepared to maintain after surgery for this patient.

**Table 1:** Representative neoadjuvant immunotherapy in resectable NSCLC.

Neoadjuvant immunotherapy model	Clinical trial	Phase	Stage	Drugs	Surgery rate	MPR	pCR
Single immunotherapy	LCMC3	II	IB-IIIB	Atezolizumab 1200 mg Q3 W	88%	21%	7%
	Checkmate-159	II	IA-IIIA	Nivolumab 3 mg/kg Q2 W	90.9%	45%	15%
Double immunotherapy	NEOSTAR	II	I-IIIA	Nivolumab 3 mg/kg Q2 W Lplilimumab 1 mg/kg Q2 W	84%	50%	38%
				Nivolumab 3 mg/kg Q2 W	84%	24%	10%
Immunotherapy combined with chemotherapy	NADIM	II	IIIA	Nivolumab 360 mg/kg Q3 W Paclitaxel plus platinum	89%	82.9%	63%
	Catherine et al.	II	IB-IIIA	Atezolizumab 1200 mg Q3 W Carboplatin and paclitaxel	97%	57%	33%
	Checkmate 816	III	IB-IIIA	Nivolumab 360 mg Q3 W Chemotherapy	83.2%	36.9%	24%
				Chemotherapy	75.4%	8.9%	2.2%

MPR, major pathological response; pCR, pathological complete response.

As we mentioned before, neoadjuvant immunotherapy or neoadjuvant immunotherapy combined with chemotherapy greatly enhanced the pCR rate in patients with resectable IB-IIIA NSCLC compared with neoadjuvant chemotherapy alone, which might change the method of neoadjuvant treatment for resectable NSCLC in the future. Partial stage IIIB and IIIC NSCLC were classified as unresectable lung cancer according to the latest NCCN guidelines. In our study, we used 2 cycles of anti-PD-1 immunotherapy combined with chemotherapy and anti-angiogenesis to successfully transform unresectable stage IIIC NSCLC into radical surgery (ypT0N0M0), which brought about the opportunity for a cure. Similarly, Huali Hu (40) also reported an N3-unresectable lung adenocarcinoma patient who received radical surgery after immunotherapy with platinum-based chemotherapy. This reminded us that IIIC patients also had an opportunity for a cure; however, selecting the “right patient” and the subsequent treatment needs more studies.

Biomarkers of neoadjuvant immunotherapy are also the focus of current research. At present, it is considered that the efficacy of neoadjuvant immunotherapy is related to the expression of PD-L1 while pathological tumor type (CheckMate 816 showed that squamous cell carcinoma has better neoadjuvant therapy efficacy than adenocarcinoma), EGFR/ALK state (the effectiveness of neoadjuvant immunotherapy in patients with EGFR/ALK mutation is poor), and more markers for predicting the treatment effect are still being explored (32, 34, 39). Our patient had a PD-L1 tumor proportion score ≥ 50%. The pathological type was squamous cell carcinoma, which may partly explain why this unresectable locally advanced NSCLC could be successfully transformed to pCR.

Antiangiogenic drugs play an antitumor role mainly by inhibiting tumor angiogenesis. Studies have shown that antiangiogenic drugs can also promote the normalization of blood vessels in tumor tissues, improve the tumor microenvironment, increase the infiltration of effector immune cells in tumor tissues, promote the arrival of chemotherapeutic drugs to tumor tissues, and cooperate with other antitumor drugs in antitumor treatment (41, 42). As mentioned earlier, the patient was unresectable at the initial diagnosis, and we used antiangiogenic agents combined with immunotherapy and chemotherapy to convert this patient to radical surgery. IMPOWER 150 demonstrates that immunotherapy combined with antiangiogenic chemotherapy has been successfully applied to patients with metastatic NSCLC. Nevertheless, the evidence-based medical evidence of neoadjuvant anti-vascular therapy is limited; whether antiangiogenic drugs can enhance the therapeutic effect of neoadjuvant immunotherapy combined with chemotherapy and their safety remain to be further explored.

Our study had some limitations. First, due to the patient’s reasons, we did not perform a biopsy on the hilar lymph nodes of the patient at initial diagnosis, but the PET-CT of the patient showed that the bilateral hilar lymph nodes were enlarged and complicated with hypermetabolism (SUVmax 9.2). Additionally, the bilateral hilar lymph nodes decreased significantly after antitumor treatment, which proves that the enlargement of left hilar lymph nodes is caused by tumor metastasis. On the other hand, studies have shown that tumor mutational burden, circulating tumor DNA, and other indicators may be related to the efficacy of immunotherapy. We did not detect these associated indicators in this patient.

## Conclusion

In conclusion, at present, neoadjuvant immunotherapy clinical research mainly includes IB-IIIA and partially resectable stage IIIB patients. We innovatively applied anti-PD-1 immunotherapy combined with chemotherapy and anti-angiogenesis therapy and successfully transformed a resectable stage IIIC NSCLC patient into radical surgery (ypT0N0M0), which brought about an opportunity for a cure and was of great significance in improving the patient’s prognosis. Our results might bring some changes to the treatment of stage IIIC lung cancer patients in the future.

## Data availability statement

The original contributions presented in the study are included in the article/supplementary material. Further inquiries can be directed to the corresponding author.

## Ethics statement

The studies involving human participants were reviewed and approved by Human and Research Ethics committees of the Renmin Hospital of Wuhan University. The patients/participants provided their written informed consent to participate in this study.

## Author contributions

All authors contributed to the study conception and design. Material preparation, data collection, and analysis were performed by GJ, SZ, XT, YH, and XL. The first draft of the manuscript was written by GJ, and all authors commented on previous versions of the manuscript. All authors contributed to the article and approved the submitted version.

## Conflict of interest

The authors declare that the research was conducted in the absence of any commercial or financial relationships that could be construed as a potential conflict of interest.

## Publisher’s note

All claims expressed in this article are solely those of the authors and do not necessarily represent those of their affiliated organizations, or those of the publisher, the editors and the reviewers. Any product that may be evaluated in this article, or claim that may be made by its manufacturer, is not guaranteed or endorsed by the publisher.
